# Endophytic fungal community of *Dysphania ambrosioides* from two heavy metal‐contaminated sites: evaluated by culture‐dependent and culture‐independent approaches

**DOI:** 10.1111/1751-7915.13308

**Published:** 2018-09-05

**Authors:** Shobhika Parmar, Qiaohong Li, Ying Wu, Xinya Li, Jinping Yan, Vijay K. Sharma, Yunlin Wei, Haiyan Li

**Affiliations:** ^1^ Medical School of Kunming University of Science and Technology Kunming 650500 China; ^2^ The First People's Hospital of Yunnan Province Kunming 650032 China; ^3^ The Affiliated Hospital of Kunming University of Science and Technology Kunming 650500 China

## Abstract

Endophytic fungal communities of *Dysphania ambrosioides,* a hyperaccumulator growing at two Pb‐Zn‐contaminated sites, were investigated through culture‐dependent and culture‐independent approaches. A total of 237 culturable endophytic fungi (EF) were isolated from 368 tissue (shoot and roots) segments, and the colonization rate (CR) ranged from 9.64% to 65.98%. The isolates were identified to 43 taxa based on morphological characteristics and rDNA ITS sequence analysis. Among them, 13 taxa (30.23%) were common in plant tissues from both sites; however, dominant EF were dissimilar. In culture‐dependent study, 1989 OTUs were obtained through Illumina Miseq sequencing, and dominant EF were almost same in plant tissues from both sites. However, some culturable EF were not observed in total endophytic communities. We suggest that combination of both culture‐dependent and culture‐independent methods will provide more chances for the precise estimation of endophytic fungal community than using either of them. The tissue had more influence on the culturable fungal community structure, whereas the location had more influence on the total fungal community structure (including culturable and unculturable). Both culture‐dependent and culture‐independent studies illustrated that endophytic fungal communities of *D. ambrosioides* varied across the sites, which suggested that HM concentration of the soil may have some influence on endophytic fungal diversity.

## Introduction

The soil is important and essential for supporting life and planetary functions such as primary production, the regulation of biogenic gases and the earth's climate, biogeochemical and water cycling, and the maintenance of biodiversity (Abhilash *et al*., 2012). Rapid and continuous worldwide industrialization, urbanization and modern agriculture practices have introduced excess of the heavy metals (HMs) into the soil. Due to their persistence in soil and their toxic nature, HMs adversely impact the ecosystem, agriculture, water quality, soil microbiota and human health (Rajkumar *et al*., 2010; Kidd *et al*., 2012; Wei *et al*., 2014). To mitigate the negative effects of HMs, the remediation of contaminated soils is gaining considerable momentum. Among all remediation methods, phytoremediation is considered as the most promising technology for its low tech‐savvy technique, cost‐effectiveness, sustainability and environment friendliness (Weyens *et al*., 2009a; Li *et al*., 2012a; Parmar and Singh, 2015). However, phytoremediation has some constraints, such as phytotoxicity, slowed plant growth, low biomass production, slow degradation of HMs, limited contaminant uptake and evapotranspiration of volatile contaminants; therefore, application of phytoremediation is limited in most circumstances (Gerhardt *et al*., 2009; Weyens *et al*., 2009b; Deng and Cao, 2017). Microbe‐assisted phytoremediation can effectively reduce this problem. For some microorganisms, it can effectively improve the plant growth by transformation of nutrient elements, production of phytohormones, or provide iron to reduce the deleterious effects of metal contamination to plants (Rajkumar *et al*., 2010).

Endophytic fungi (EF) can be defined as fungi that reside asymptomatically in the interior of host plant tissues (Hyde and Soytong, 2008). They are ubiquitous in nature and have been successfully isolated from wide range of hosts belonging to a wide range of environmental conditions (Redman *et al*., 2002; Bashyal *et al*., 2005; Rosa *et al*., 2009; Mishra *et al*., 2012). It was estimated that there are at least one million species of EF worldwide (Ganley *et al*., 2004). Interestingly, diverse EF are also found in highly HM contaminated environments (Xiao *et al*., 2010; Deng *et al*., 2011; Choo *et al*., 2015; Yamaji *et al*., 2016), and recent advances suggest that the EF can enhance HMs accumulation and tolerance capacity of host plants (Khan and Doty, 2011; Li *et al*., 2012c; Shen *et al*., 2013; Yamaji *et al*., 2016). The possible mechanism of increased tolerance to HM stress in the host plant by endophytes involves enhancements of antioxidative system, changing HM distribution in plant cells and detoxification of HM (Wang *et al*., 2016).

*Dysphania ambrosioides* (L.) Mosyakin & Clemants, previously known as *Chenopodium ambrosioides*, is an invasive plant in China. Previous studies have indicated that it is a dominant plant species in some Pb–Zn contaminated sites in Huize County, Yunnan Province, Southwest China (Li *et al*., 2012c, 2016), and it was reported as a Pb‐hyperaccumulator (Wu *et al*., 2004). Our previous studies have revealed that *D. ambrosioides* growing in Pb–Zn contaminated locations have high diversity of EF, and some of them showed better Pb, Zn, Cd tolerance and could enhance host plant growth and affect its HMs accumulation (Li *et al*., 2016; Sun *et al*., 2018). To find out the role of endophytes in host plants’ HM adaptation and explore them in phytoremediation, the understanding of endophytic community is critical. Recent advances in the modern molecular phylogenetic and high‐throughput DNA sequencing have provided an inclusive method to study culture‐independent microbial community (Shakya *et al*., 2013; Senés‐Guerrero and Schüßler, 2016; De Corte *et al*., 2018). Comparison of the abundance of culturable and unculturable endophytes by direct sequencing of *Deschampsia flexuosa* well established that unculturable endophytes are common and potentially more abundant than the culturables (Tejesvi *et al*., 2010). In this study, we aim to investigate both the culturable and total (including culturable and unculturable) endophytic fungal community of *D. ambrosioides* collected from two HM‐contaminated through culture‐dependent and culture‐independent approaches.

## Results

### Plant and soil properties

The two investigated sites were situated in the area where Pb–Zn mining has been carrying out for more than 300 years. Consistent with this, soils and plants from both sampling sites were heavily polluted by Pb, Zn and Cd (Table 1) (GB13106‐1991, 1991; GB15618‐1995, 1995; GB2762‐2012, 2012). Overall, the level of HMs in the soils from the slag heap was comparatively higher than that from the wasteland. However, the bio‐available (DTPA‐TEA extractable fractions) concentrations of HMs in the soils from the slag heap were significantly lower (*P *<* *0.05) than that from the wasteland. Consistent with this, the level of HMs in the plants from the slag heap was significantly lower (*P *<* *0.05) than that from the wasteland. Except the available P, other soil physico‐chemical characteristics, that is organic matter, total N, P, K, the available K and hydrolysable N, of the soils from the slag heap were significantly lower than those of the soils from the wasteland (Table 2).

**Table 1:** Heavy metal content of plants and soils (mean ± SD)

Sample site	Plants (mg kg^−1^, dry weight)			Soils (mg kg^−1^, dry weight) Total HM			Soils (mg kg^−1^, dry weight) Bio‐available HM		
	Pb	Zn	Cd	Pb	Zn	Cd	Pb	Zn	Cd
Slag heap	52.95 ± 0.68^a^	1648.93 ± 24.65^a^	27.12 ± 0.67^a^	4276.18 ± 61.27^a^	18496.89 ± 1357.70^a^	8.89 ± 0.10^a^	177.38 ± 23.78^a^	179.54 ± 35.69^a^	0.49 ± 0.12^a^
Wasteland	264.13 ± 5.88^b^	21436.25 ± 388.98^b^	48.49 ± 1.55^b^	4126.37 ± 57.16^a^	7127.27 ± 143.42^b^	7.45 ± 0.14^b^	1020.24 ± 29.74^b^	1165.31 ± 121.24^b^	3.30 ± 0.1^b^
Standard	0.3^c^	20^d^	0.2^c^	500^e^	500^e^	1^e^			

Different letters in the same column indicate a significant difference at *P *<* *0.05.

^c^The standard of ‘national food safety standards’ (GB2762‐2012).

^d^The standard of ‘tolerance limit of zinc in foods’ (GB13106‐1991).

^e^The standard of ‘environment quality standard for soils’(GB 15618‐1995, grade III), pH > 6.5.

**Table 2:** Physico‐chemical characteristics of soils

Sample site	pH	Organic matter	Total N	Total P	Total K	Hydrolysable N	Available P	Available K
		(g kg^−1^, dry weight)				(mg kg^−1^, dry weight)		
Slag heap	7.79	37.79 ± 0.35^a^	0.77 ± 0.14^a^	1.08 ± 0.03^a^	3.1 ± 0.15^a^	42.75 ± 9.32^a^	21.01 ± 4.88^a^	113.93 ± 9.36^a^
Wasteland	6.19	109.29 ± 0.36^b^	1.57 ± 0.03^b^	2.03 ± 0.11^b^	4.03 ± 0.15^b^	88.63 ± 1.08^b^	18.75 ± 1.56^a^	181.43 ± 6.87^b^

Mean ± standard deviation from three replicates. Different letters in the same column indicate a significant difference at *P *<* *0.05.

### Culturable endophytic fungal community

**Figure 1:**
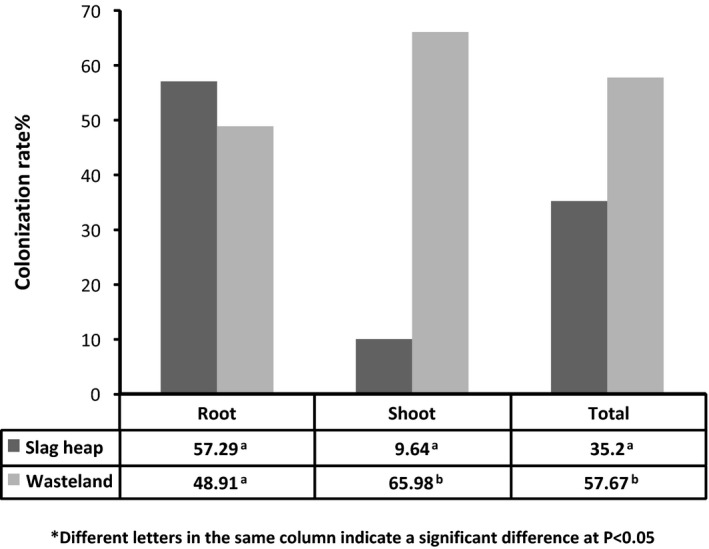
Colonization rate (CR%) of the endophytic fungi of *D. ambrosioides* from two sites.

A total of 237 EF were isolated from 368 tissue segments of *D. ambrosioides* growing naturally in the slag heap and wasteland (Table 3). The colonization rate (CR) was calculated as the total number of plant segments infected by one or more fungi divided by the total number of segments incubated (Sun *et al*., 2011). The colonization rate (CR) of different plant tissues from two sites ranged from 9.64% to 65.98% (Fig. 1). The total CR of plants from the slag heap (35.2%) was significantly (*P *<* *0.05, chi‐square test) lower than that of the plants from the wasteland (57.67%; Fig. 1). Similarly, the CR of the shoots from the slag heap (9.64%) was significantly lower (*P *<* *0.05, chi‐square test) than that of the shoots from the wasteland (65.98%). However, the CR of the roots from the slag heap (57.29%) was a little higher than that of roots from the wasteland (48.91%; *P *>* *0.05, chi‐square test) (Fig. 1).

**Table 3:** Taxa, number of isolates (I) recovered, relative frequency (RF%) and Shannon index (*H′*) of endophytic fungi of *D. ambrosioides* from two sites

Taxa	Plants from the slag heap						Plants from the wasteland						Grand total	
	Shoot		Root		Total		Shoot		Root		Total			
	I	RF%	I	RF%	I	RF%	I	RF%	I	RF%	I	RF%	I	RF%
*Alternaria* sp.	2	25.00	0	0	2	2.41	18	19.35	0	0	18	11.69	20	8.44
*Alternaria tenuissima*	0	0	0	0	0	0	2	2.15	0	0	2	1.30	2	0.84
*Aposphaeria* sp.	0	0	1	1.33	1	1.20	0	0	0	0	0	0	1	0.42
Ascomycota sp.	0	0	0	0	0	0	1	1.08	0	0	1	0.65	1	0.42
*Ceotrichum* sp.	0	0	0	0	0	0	2	2.15	0	0	2	1.30	2	0.84
*Cephalosporium* sp. 1	0	0	3	4.00	3	3.61	0	0	0	0	0	0	3	1.27
*Chaetomium globosum*	0	0	0	0	0	0	2	2.15	3	4.92	5	3.25	5	2.11
*Chrysosporium lobatum*	0	0	2	2.67	2	2.41	1	1.08	0	0	1	0.65	3	1.27
*Cladosporium* sp. 1	0	0	5	6.67	5	6.02	5	5.38	6	9.84	11	7.14	16	6.75
*Cladosporium* sp. 2	0	0	9	12.00	9	10.84	0	0	0	0	0	0	9	3.80
*Colletotrichum* sp. 1	0	0	1	1.33	1	1.20	0	0	7	11.48	7	4.55	8	3.38
*Colletotrichum* sp. 2	0	0	2	2.67	2	2.41	0	0	0	0	0	0	2	0.84
*Dendryphion* sp.	0	0	1	1.33	1	1.20	1	1.08	0	0	1	0.65	2	0.84
*Diplodia* sp.	0	0	0	0	0	0	0	0	2	3.28	2	1.30	2	0.84
*Discosia* sp.	0	0	1	1.33	1	1.20	0	0	0	0	0	0	1	0.42
*Epicoccum nigrum*	0	0	5	6.67	5	6.02	0	0	0	0	0	0	5	2.11
*Fusarium* sp. 1	0	0	3	4.00	3	3.61	0	0	2	3.28	2	1.30	5	2.11
*Fusarium* sp. 2	0	0	1	1.33	1	1.20	0	0	1	1.64	1	0.65	2	0.84
*Fusarium* sp. 3	0	0	0	0	0	0	0	0	2	3.28	2	1.30	2	0.84
*Fusarium* sp. 4	0	0	1	1.33	1	1.20	0	0	0	0	0	0	1	0.42
*Fusarium* sp. 5	0	0	1	1.33	1	1.20	0	0	0	0	0	0	1	0.42
*Fusarium* sp. 6	0	0	1	1.33	1	1.20	0	0	0	0	0	0	1	0.42
*Gilmaniella* sp.	1	12.50	0	0	1	1.20	0	0	0	0	0	0	1	0.42
*Hainesia* sp.	0	0	0	0	0	0	0	0	1	1.64	1	0.65	1	0.42
*Humicola fuscoatra*	0	0	0	0	0	0	0	0	4	6.56	4	2.60	4	1.69
*Ilyonectria radicicola*	0	0	0	0	0	0	0	0	9	14.75	9	5.84	9	3.80
*Macrophoma* sp.	0	0	2	2.67	2	2.41	0	0	1	1.64	1	0.65	3	1.27
*Micropora* sp.	0	0	0	0	0	0	7	7.53	0	0	7	4.55	7	2.95
*Monilia* sp.	0	0	2	2.67	2	2.41	1	1.08	1	1.64	2	1.30	4	1.69
*Monocillium* sp.	0	0	1	1.33	1	1.20	0	0	0	0	0	0	1	0.42
*Mucor* sp.	0	0	3	4.00	3	3.61	0	0	0	0	0	0	3	1.27
*Nodulisporium* sp.	1	12.50	0	0	1	1.20	1	1.08	0	0	1	0.65	2	0.84
*Penicillium* sp.	0	0	0	0	0	0	0	0	4	6.56	4	2.60	4	1.69
*Peyronellaea* sp.	2	25.00	2	2.67	4	4.82	15	16.13	4	6.56	19	12.34	23	9.70
*Phoma* sp.	1	12.50	4	5.33	5	6.02	29	31.18	2	3.28	31	20.13	36	15.19
*Phomopsis columnaris*	0	0	0	0	0	0	1	1.08	5	8.20	6	3.90	6	2.53
*Plectosphaerella* sp.	1	12.50	14	18.67	15	18.07	0	0	2	3.28	2	1.30	17	7.17
*Rhynchophoma* sp.	0	0	0	0	0	0	2	2.15	1	1.64	3	1.95	3	1.27
*Septoria* sp.	0	0	0	0	0	0	3	3.23	0	0	3	1.95	3	1.27
*Verticillium* sp.	0	0	7	9.33	7	8.43	0	0	0	0	0	0	7	2.95
Unidentified 1	0	0	0	0	0	0	2	2.15	0	0	2	1.30	2	0.84
Unidentified 2	0	0	3	4.00	3	3.61	0	0	0	0	0	0	3	1.27
Unidentified 3	0	0	0	0	0	0	0	0	4	6.56	4	2.60	4	1.69
Total	8	100	75	100	83	100	93	100	61	100	154	100	237	100
Shannon *H′*	1.733		2.823		2.927		2.143		2.724		2.823		3.233	
Simpson 1−*D*	0.813		0.919		0.926		0.827		0.922		0.911		0.940	

**Figure 2:**
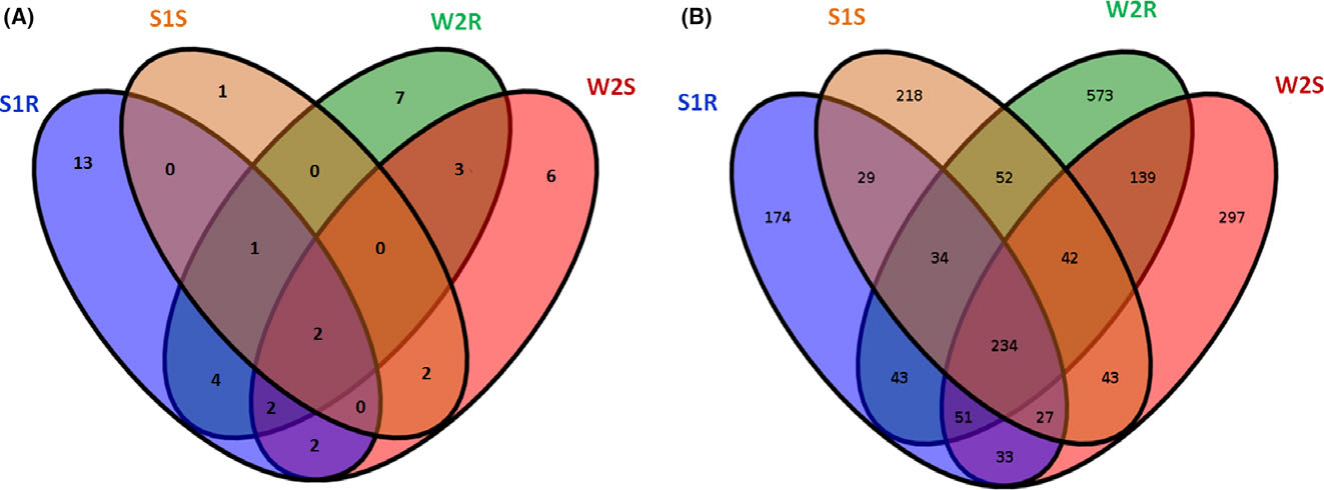
Venn diagram of the culturable endophytic fungi (A) and of the OTUs (B) of *D. ambrosioides* from two sites (S1 = Slag heap, W2 = Wasteland; S = Shoot, R = Root).

The endophytic fungal isolates were identified to 43 taxa based on morphological characteristics and ITS sequence analysis (Table 3), and the rDNA ITS sequences of the fungi subjected to molecular identification in this study were deposited in GenBank (Accession numbers are KT291413, KT291414, KT291415, KT291416, KT291418, KT291419, KT291420, KT291422, KT291423, KT291428, KT291426 and KT291432). Among them, 13 taxa (30.23%) co‐existed in plants from both sites, with a total of 27 and 29 taxa recorded in plants from the slag heap and wasteland respectively (Fig. 2a, Table 3). The relative frequency (RF) was calculated as the number of isolates of one species divided by the total number of isolates (Yuan *et al*., 2011a). The dominant EF of plants from the slag heap were *Plectosphaerella* sp., *Cladosporium* sp. 2 and *Verticillium* sp., showing RF of 18.07%, 10.84% and 8.43% respectively. The dominant EF of plants from the wasteland were *Phoma* sp., *Peyronellaea* sp., *Alternaria* sp. and *Cladosporium* sp. 1, showing RF of 20.13%, 12.34%, 11.69% and 7.14% respectively (Table 3).

**Figure 3:**
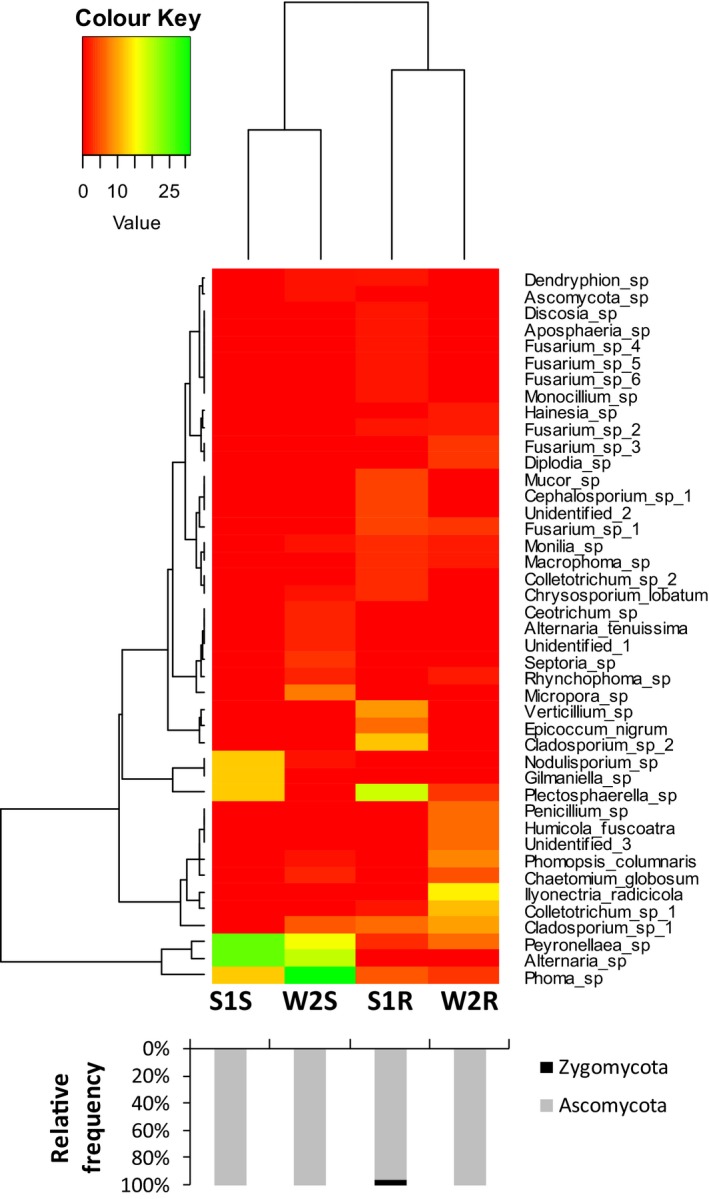
Heat maps of the relative abundance of culturable fungal endophytes of *D. ambrosioides* in different tissues and locations. Different colour means the different RF of the taxa in the all four samples (green means high RF) (S1 = Slag heap, W2 = Wasteland; S = Shoot, R = Root). The black and grey bars below show the phylum of the fungal endophytes isolated.

The endophytic fungal community of shoots from two locations was clustered together, and similarly that of the roots together (Fig. 3). The endophytic fungal diversity was evaluated using the Shannon index (*H′*), which has two main components, evenness and the number of species (Spellerberg and Fedor, 2003). The Simpson index (1 − *D*) estimates the probability that two randomly selected individuals from a community belong to different species (Simpson, 1949). The *H′* and Simpson (1 − *D*) of EF from the slag heap and wasteland were 2.927 and 2.823, and 0.926 and 0.911 respectively (Table 3).

### Total endophytic fungal community

A dataset was developed that consisted of 46 172 filtered high‐quality and classified unique fungal ITS2 gene tags with a maximum length of 442 bp and minimum length of 238 bp (Table 4). All the sequences were clustered with the representative sequences, and >97% sequence identity cut‐off was used; all tags of ITS2 region were classified at each level. The number of operational taxonomic units (OTUs) per sample ranged from 625 to 1168 (Table 4). In the plants from the slag heap, the OTUs of roots (625) were less abundant than that of shoots (679); however, in the plants from the wasteland, the OTUs of roots (1168) were more abundant than that of shoots (866) (Table 4).

**Table 4:** The unique tags and α diversity of endophytic fungi from *D. ambrosioides* (distance < 0.03)

Sample site	Sample ID	Unique tags	Number of OTU	α Diversity		
				Chao 1	Shannon	Simpson
Slag heap	S1S	3807	679	1457.010	4.462	0.038
	S1R	4542	625	1287.625	4.066	0.062
Wasteland	W2S	12 380	866	2033.481	3.323	0.095
	W2R	25 443	1168	2486.037	3.149	0.093

R = Root; S = Shoot; S1 = Slag heap, W2 = Wasteland.

**Figure 4:**
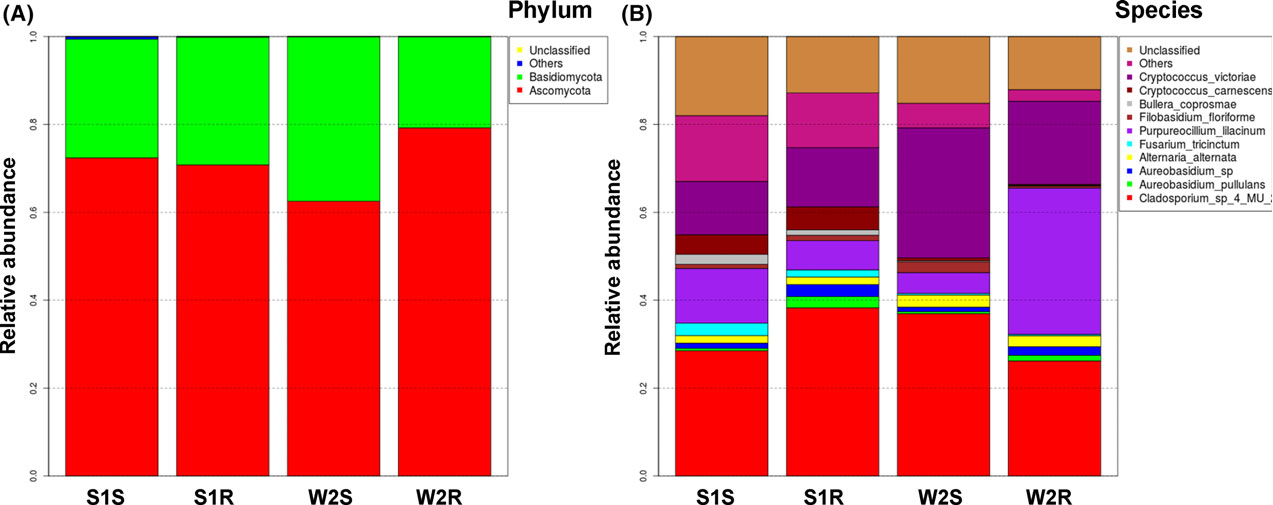
Relative abundance of the different taxonomic units of endophytic fungi (species and phylum) identified in *D. ambrosioides* samples from two sites (S1 = Slag heap, W2 = Wasteland; S = Shoot, R = Root).

**Figure 5:**
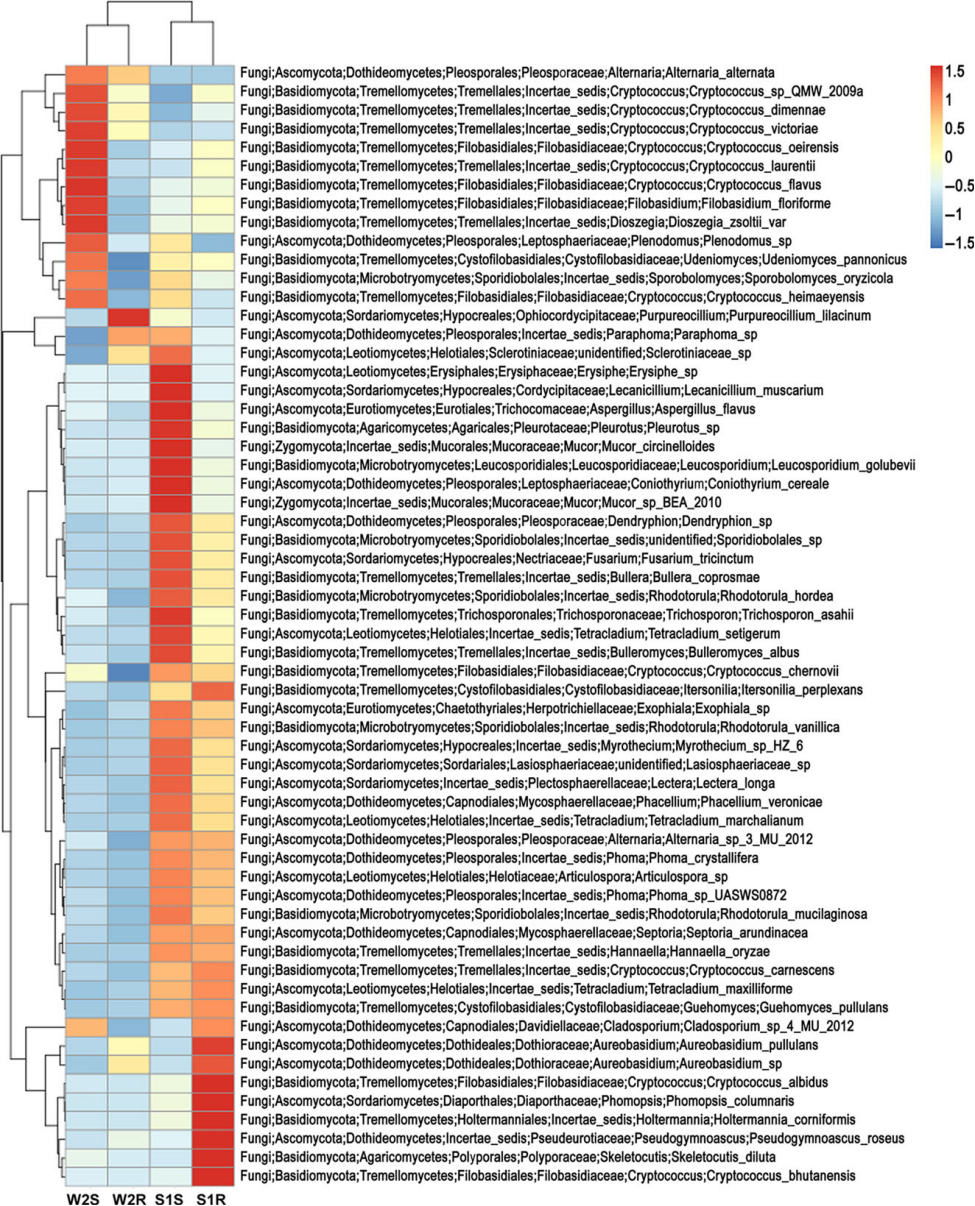
Heat maps of the relative abundance of the total fungal endophytes of *D. ambrosioides* in different tissues and locations. Different colour indicates difference in relative abundance (Log_10_) of the taxa in all four samples (S1 = Slag heap, W2 = Wasteland; S = Shoot, R = Root).

The OTUs were analysed at different taxonomic level. Ascomycota was found to be the most abundant (62.5% to 79.2%) across all the samples analysed, followed by Basidiomycota (20.7% to 37.4%), while Chytridiomycota and Zygomycota were found to be rare and incidental (Fig. 4a). The relative abundance of *Cladosporium* sp. was recorded to be highest across all the samples followed by *Cryptococcus victoriae* and *Purpureocillium lilacinum* (Fig. 4b). Other abundant species recorded were *Aureobasidium pullulans*,* Aureobasidium* sp., *Alternaria alternate*,* Fusarium tricinctum*,* Filobasidium floriforme*,* Bullera coprosmae* and *Cryptococcus carnescens*, while relatively large number (>12%) of species were remained unclassified (Fig. 4b). The relative abundance of the total EF of *D. ambrosioides* in different tissues and locations was shown in the heat maps (Fig. 5). It was observed that the location had more influence over tissue on the community structure of EF, as shoots and roots of plants from the slag heap clustered together, and in the same way shoots and roots of plants from the wasteland (Fig. 5).

The computational analysis of α‐diversity estimated the richness and diversity of plant tissues from two sites at OTU cut‐offs of 0.03 distance units (Table 4). Among them, Chao1 estimated minimum number of OTUs, and inverse Simpson diversity index indicated the richness of the communities (Akinsanya *et al*., 2015). Shannon index used in this study was as an expression or index of some relation between number of species and number of individuals (Spellerberg and Fedor, 2003). It was found that both the Chao1 and Simpson diversity of the shoots and roots of the plants from the slag heap were significantly lower (*P *<* *0.05) than that of plants from the wasteland (Table 4). However, the Shannon indices (*H*′) of the shoots and roots of plants from the slag heap were significantly higher than that of plants from the wasteland (Table 4). Beta diversity analysis indicated that the microbial structures of the shoots and roots of plants from slag heap clustered to one group, while the shoots and roots of plants from the wasteland clustered to another group, same as that observed in the heat maps (Fig. 5).

The raw sequencing data generated from this study have been deposited in NCBI SRA (http://www.ncbi.nlm.nih.gov/sra) under the accession number SRA510221.

## Discussion

The colonization rate (CR) of culturable endophytic fungi (EF) of *D. ambrosioides* from two sites was 35.2% and 57.67% respectively. They were significantly lower than those reported in other environments without HM stress, which were usually ranged from 95% to 100% (Gambo and Bayma, 2001; Arnold *et al*., 2007; Rhoden *et al*., 2012). In addition, it was found that although the two sampling sites were close to each other (1.5 to 2 km), and the environmental conditions were similar except for soil physico‐chemical characteristics (Tables 1 and 2); however, the CR of EF of plants from the wasteland was significantly higher than that of the slag heap (Fig. 1). Moreover, the dominant genera of culturable EF of *D. ambrosioides* from two sites were different, too. In the slag heap, the dominant EF were *Plectosphaerella* sp., *Cladosporium* sp. 2 and *Verticillium* sp., while, in the plants from the wasteland, the dominant EF were *Phoma* sp., *Peyronellaea* sp., *Alternaria* sp. and *Cladosporium* sp. 1 (Table 3). This difference may be due to the difference in the HM concentration of the soils at two sites (Table 1). The result was consistent with previous studies that the endophytic diversity can vary with the level of the pollution (Helander *et al*., 1993; Li *et al*., 2012c; Schmidt *et al*., 2018).

**Figure 6:**
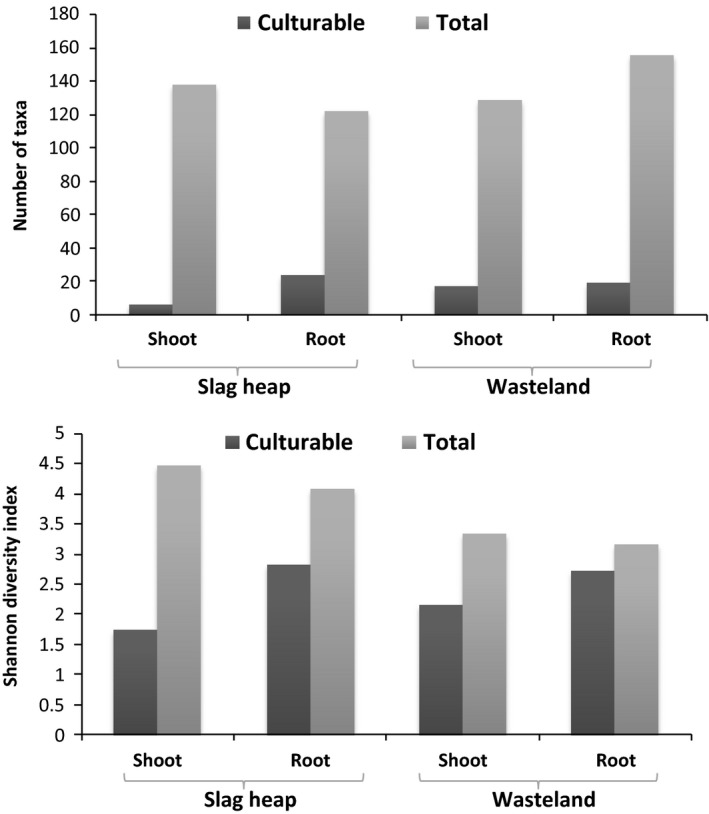
Comparison of the isolated and detected taxa and diversity of culturable and total endophytic fungal communities.

Four fungal phyla were recovered by culture‐independent method, while only two fungal phyla (Ascomycota, Zygomycota) were detected by culture‐dependent method. Ascomycota was found to be the most common EF in plants by both culture‐dependent and culture‐independent methods. This is consistent with previous finding that Ascomycota was the dominant group in soils, marine environments, mangroves and endophytic community (Gazis and Chaverri, 2010; Peršoh *et al*., 2010; Simões *et al*., 2015; Khan *et al*., 2017). Forty‐three taxa were recovered of culturable endophytes, while 1989 OTUs were obtained in Illumina Miseq sequencing, which supported the fact that only a very small portion of endophytes can be cultured (Fig. 6) (Torsvik and Øvreås, 2002; Alain and Querellou, 2009). In addition, culture‐dependent study showed that the diversity of EF of roots was higher than that of shoots at both sites; however, the results of the culture‐independent method were reverse (Fig. 6). Moreover, the diversity of total EF of both shoots and roots from the slag heap was higher than that of plants from the wasteland, which was also different from the results of culture‐dependent method (Fig. 6). These results suggested that to understand the endophytic community comprehensively, combination of both culture‐dependent and culture‐independent methods are necessary.

The culturable EF of shoots from two locations clustered to one group, while the EF of roots from two locations clustered to another group, which suggested that the tissue has more influence on the endophytic fungal community than the location (Fig. 3). But contrary to this, the results of the culture‐independent method indicated that the location has more influence on the endophytic fungal community than the tissue (Fig. 5). Beta diversity analysis and Bray–Curtis cluster analysis also supported this inference. As environmental parameters of two locations were almost similar except the soil physico‐chemical characteristics (Tables 1 and 2), therefore, we suggest that the HM concentration of the soil may have some influence on the endophytic fungal diversity. This is consistent with previous findings that the metal contamination and other pollutants can affect the fungal diversity and community structure (Danti *et al*., 2002; Op De Beeck *et al*., 2015; Glynou *et al*., 2016).

The dominant EF of different plant tissues from two locations found by culture‐independent method were almost the same (Fig. 4b). However, the dominant culturable EF of *D. ambrosioides* differed with the locations (Table 3). Despite this, some genera were found to be dominant EF in both culture‐dependent and culture‐independent studies, such as *Cladosporium* sp. and *Alternaria* sp. (Table 3, Fig. 4b). Contrary to this, some culturable EF were not detected in culture‐independent study, such as *Chaetomium* and *Macrophoma*. This may be repercussion of the low ratio of these fungi in the plants and thus were possibly below the detection level of Illumina Miseq sequencing. However, they were recovered profusely on artificial media for fast‐growing characteristics (Mohamed *et al*., 2010). The same phenomenon was observed in other works (Premalatha and Kalra, 2013).

There were 12% to 17.98% OTUs that remained unclassified (Fig. 4). In a previous study, the endophytic fungal assemblage in stems of wild rice in China was characterized using a combination of morphology and molecular techniques and observed that 30% of the total taxa recovered remained sterile and unidentifiable (ITS sequence similarity 83%–94%) even to the genus level (Yuan *et al*., 2011b). There could be two reasons for this: first and foremost, the unidentified species may represent lineages new to the fungal biota, and second, a large number of species are undescribed and uncharacterized in the database or size of the OTUs was not enough for the 100% query coverage. These endophytes need more attention in the future study.

Plants in association with microbes can be applied to remove the labile/bio‐available pool of inorganic contaminants from a site, remove or degrade organic contaminants, stabilize or immobilize contaminants (Megharaj and Naidu, 2017). The associated microbes can enhance host plant growth, increase solubility and bioavailability of contaminant and alter heavy metal accumulation through IAA, siderophores, organic acids and biosurfactant production (Li *et al*., 2012a; Ullah *et al*., 2015a,b; Tirry *et al*., 2018). Moreover, microbes can indirectly enhance phytoremediation by stimulating soil microbial communities (Burges *et al*., 2017). Endophytes have been demonstrated to play a key role in host plant adaptation to polluted environments and that they can enhance phytoremediation by mobilizing/degrading or immobilizing contaminants in the soil, promoting plant growth, decreasing phytotoxicity and improving plants’ metal tolerance, as well as in other ways (Li *et al*., 2012a). Therefore, endophytes of *D. ambrosioides* may also have enhanced host plant phytoremediation. Future experiments will be necessary to further evaluate the roles and the mechanisms of the endophytes.

## Experimental procedures

### Description of site and sampling

The investigated sites were situated in Zhehai, Huize County, Yunnan Province, Southwest China (25°48′–27°04′N, 103°03′–103°55′E), where Pb–Zn mining has been carrying out for more than 300 years. The region belongs to temperate monsoon climate, with short, mild, dry winters and warm, rainy summers. The average elevation is 2099 m, and the annual mean temperature and annual rainfall are 12.6°C and 847.1 mm respectively. The frost‐free period lasts for approximately 202 days. This region is full of slag heaps and wastelands, which are covered with sparse vegetation. *D. ambrosioides* was one of the dominant plant species at the sampling sites (Qin *et al*., 2013).

Healthy plants of *D. ambrosioides* were collected from both slag heap and wasteland sites that were about 1.5–2 km apart from each other. At each sampling location, randomly 15 healthy *D. ambrosioides* plants were collected with each plant at least 30 m apart from another, and the adjacent soils were also collected at a depth of 5–10 cm and mixed thoroughly. All the collected samples were immediately stored in a sterile polythene bags, labelled accordingly and brought to the laboratory under refrigerated conditions. The plant samples were processed within 24 h for the isolation of endophytic fungi and total DNA extraction.

### Physico‐chemical characteristics and heavy metal concentration analysis

Soil samples were air‐dried (25°C) and then crushed and sieved with a 0.15‐mm mesh to get fine powders. Then, soil organic matter, total nitrogen, total phosphorus, total potassium, alkaline hydrolysable nitrogen, available phosphorus, available potassium and pH (soil: H_2_O = 1:2.5) were measured according to previously described methods (Lin *et al*., 2017). For HM concentration analysis, plant samples were washed with distilled water to remove surface element trace and then were divided into roots and shoots and oven‐dried at 65°C for 48 h until constant weight was achieved. Subsequently, the samples were crushed to fine powders with a mortar and pestle, and 0.2 g roots/shoots powders were digested with 5 ml HNO_3_ (65% w/w) at 110°C for 2 h and then cooled and added with 1 ml H_2_O_2_ (30% w/w) and heated for 1 h. Finally, the digests were diluted to 50 ml with triple deionized water in a volumetric flask (Shen *et al*., 2013). Soil powders (0.5 g) were digested with 4 ml HCl–HNO_3_ (3:1, v/v) mixture at 80°C for 30 min, then 100°C for 30 min, finally 120°C for 1 h. Thereafter, cooled and 1 ml HClO_4_ was added to continue digesting at 100°C for 20 min, followed by 120°C for 1 h. Finally, the digests were diluted to 50 ml with triple deionized water in a volumetric flask. The concentrations of bio‐available Pb, Zn and Cd in soils were extracted by diethylenetriaminepentaacetic acid‐triethanolamine (DTPA‐TEA) (Huang *et al*., 2006). All the samples were prepared in triplicates. The concentrations of Pb, Zn and Cd in plant and soil digests were determined by flame atomic absorption spectrometry (Li *et al*., 2014).

The mean and standard deviation of the HM concentrations was calculated using three replicates of a mixed plant and soil samples from two sites respectively. A t‐test was performed to determine the differences in mean HM concentration between samples from the two sites, and *P* was set at <0.05.

## Endophytic fungal community of *D. ambrosioides*

The fungal endophytic communities in different tissues of *D. ambrosioides* from two sites were evaluated using both cultivation‐dependent and cultivation‐independent approaches.

### Surface sterilization of plant tissues

The plants were washed with running tap water to remove the adhered soil particles and other contaminants. Thereafter, five leaves, five stems and five roots segments (about 6 cm long) were randomly selected from each plant, and thus, overall 75 leaves, 75 stems and 75 roots fragments were selected from the sampled plants from each site. The surface sterilization was carried out by sequentially dipping the fragments in 75% ethanol for 2 min, followed by 5% sodium hypochlorite for 2 min and finally 3–5 times rinsed with sterile distilled water (Li *et al*., 2012b). The surface sterilized fragments were dried on sterilized filter paper, and the efficacy of the surface sterilization process was confirmed by making imprints of disinfected plant fragments on Petri dish containing PDA (potato dextrose agar); the absence of any fungal growth was observed as an effective surface sterilization (Schulz *et al*., 1998).

## Culturable endophytic fungal community

### Fungal endophytes isolation and identification

The surface sterilized fragments were cut down to small segments of 0.5 × 0.5 mm using a sterile blade under aseptic conditions. Then, 100 root segments and 100 shoot segments (50 stems and 50 leaves) from each sampling site were placed on Petri dishes containing PDA supplemented with 0.5 g l^−1^ streptomycin sulphate. The plates were incubated at 25 ± 1°C and checked every alternate day for 45 days; the emerging fungal mycelia from the plant tissues were transferred to fresh PDA plates. All the isolates were deposited in Medical School of Kunming University of Science and Technology.

Fungal morphological identification was based on the morphology of the colony as well as the mechanism of spore production and spore characteristics (Barnette and Hunter, 1987; Ellis, 1988). For frequently occurring morphotypes that were either sterile or sporulating structures that were difficult to identify to genus level, molecular identification was attempted using the internal transcribed spacer (ITS) region and a reference database. A total of 61 isolates from 12 morphotypes were selected to conduct molecular analysis. To produce fresh biomass of pure mycelium for DNA extraction, isolates were transferred to fresh PDA plates and incubated at 25°C for 1–2 weeks. Then, some mycelium were scraped off and DNA was extracted using PowerSoil^®^ DNA Isolation Kit (Mobio: Carlsbad, California, USA) and amplified with the primers ITS1 and ITS4 (Khan and Lee, 2013). PCR products were purified using Cycle‐pure Kit (Bioteke: Beijing, China) according to the manufacturer's protocol and were sent to Sangon Biotech Co., Ltd. (Shanghai, China) for sequencing. Finally, the sequences obtained in this study were uploaded to GenBank database (http://www.ncbi.nlm.nih.gov/) and the similarities of them with the published sequences in GenBank database were determined by blast.

### Data analysis

The colonization rate (CR) corresponds to the number of endophytic fungi colonized inside host plants and was calculated as the total number of plant segments infected by one or more fungi divided by the total number of segments incubated (Sun *et al*., 2011). The relative frequency (RF) was calculated as the number of isolates of one species divided by the total number of isolates (Yuan *et al*., 2011a). The Shannon index (*H′*) was calculated according to the following formula: $H^{\prime }=\sum_{i=1}^{k}P_{i}\times \ln P_{i}$, where *k* is the total species number of one plot and *P*_*i*_ is the relative abundance of endophytic fungal species in one plot (Spellerberg and Fedor, 2003). Simpson index (1 − *D*) was calculated according to the following formula: 1 − [*D* = ∑(*n*_*i*_/*n*)^2^] where *n*_*i*_ is the number of distinct species (*i*) and (*n*) is the abundance of each species in the community (Simpson, 1949).

spss software ver. 17.0 was used for statistical analysis. Chi‐squared test was used to compare the differences in the CR of endophytes from two sites.

## The total endophytic fungal community

### Total genomic DNA extraction and sequencing

The surface sterilized root and shoot segments were transferred to sterilized mortars and homogenized in liquid nitrogen individually. The total genomic DNA was extracted from approximately 0.2 mg powdered sample using PowerSoil^®^ DNA Isolation Kit (Mobio, USA) and was verified by gel electrophoresis (1% agarose, 120 V, 30 min) (Khan and Lee, 2013). The fragment of the ITS2 region of about 360 bp length was targeted using a forward primer (5′‐GATGAAGAACGYAGYRAA‐3′) combined with a reverse primer (5′‐TCCTCCGCTTATTGA TATGC‐3′) for fungal community analysis. The PCR contained 2.5 μl Takara 10 × Ex Taq Buffer (Takara, China), 1.5 μl Mg^2+^ (25 mM MgCl_2_), 2 μl dNTP Mixture (2.5 mM each), 0.25 μl Takara Ex Taq DNA Polymerase (2.5 units/μl), 1 μl Template DNA (20 ng), 0.5 μl forward primer (10 μM), 0.5 μl reverse primer (10 μM) and 16.75 μl Sterilized ddH_2_O in a volume of 25 μl. PCR amplification was performed in a thermal cycler with the following cycling parameters: an initial denaturation at 94°C for 2 min, followed by 34 cycles of denaturation at 94°C for 30 s, annealing at 57°C for 30 s, extension at 72°C for 30 s and a final extension at 72°C for 5 min. The amplified PCR products were verified by electrophoresis in a 1% (wt/vol) agarose gel and visualized under a UV transilluminator. DNA band with the expected size was excised from the agarose gel with a clean and sharp scalpel. The PCR products were purified from agarose gels using a QIAGEN Plasmid Mega Kit 25 (Qiagen: Hilden, Germany) according to the manufacturer's protocol. The DNA concentration was measured in a micro‐spectrophotometer ND‐1000 (NanoDrop Technologies: Wilmington, USA), after which equimolar concentrations of the barcoded amplicons were collected per library and diluted to 100 ml using TE buffer. The library was bi‐directionally sequenced using an Illumina MiSeq Desktop at Guangzhou Gene Denovo Biological Technology Company (Guangzhou, China). The sequence analysis was carried out using the blast algorithm in GenBank nucleotide database (http://www.ncbi.nlm.nih.gov/blast/).

### Sequencing data analysis

The raw Illumina Miseq sequencing data were obtained in FASTA files along with sequencing quality files. The files were accessed using mothur v.1.34.0 bioinformatics software (Schloss *et al*., 2009) for further processing and analyses (Schloss *et al*., 2011). All sequences were denoised before barcodes, and primers were removed. The cleaned‐up sequences were aligned and classified along known sequences in the SILVA rRNA database (Pruesse *et al*., 2007). Chimeric sequences together with known mitochondria and chloroplast sequences were filtered, and the remaining sequences were assigned to operational taxonomic units (OTUs) based on a 97% similarity criterion. Rarefaction curves of the OTUs were performed to check the sample adequacy using a 50 sequence increment. The representative sequence for each OTU was provided taxonomical annotation by Naive Bayesian based classifier, the RDP classifier [http://rdp.cme.msu.edu/classifier/classifier.jsp] at 0.5 confidence threshold (Wang *et al*., 2007).

To indicate the microbial diversity in plant tissues, the α‐diversity indices (including Chao1, Simpson and Shannon indices) were quantified in terms of OTU richness. OTU expression spectrum image was performed using r software. Subsequently beta diversity analysis was carried out calculating Whittaker's using formula β = ((*S* ÷ α) − 1), where *S* represents the total number of OTU in two samples and α represents the total of OTU of the two samples (Whittaker, 1960). The hierarchical clustering of Bray–Curtis was carried out with r software.

## Conclusion

High‐throughput sequencing study demonstrated that *D. ambrosioides* harbour more EF than culture‐dependent method can estimate, and the location has more impact on the endophytic fungal community than the tissue type. The dominant EF of plants from two locations were almost similar: *Cladosporium* sp. was the most dominant EF followed by *Cryptococcus victoriae* and *Purpureocillium lilacinum*. However, the dominant culturable EF of *D. ambrosioides* differed with the locations. Although Ascomycota was observed to be the most dominant phylum followed by Basidiomycota in both culture‐dependent and culture‐independent studies, however, Chytridiomycota and Zygomycota were only observed in culture‐independent studies. On the contrary, some culturable EF were not detected by culture‐independent method. We suggest that the combination of culture‐dependent and culture‐independent methods more precisely reveals the structure of the endophytic fungal community than does either method alone. Both culture‐dependent and culture‐independent studies illustrated that endophytic fungal communities of *D. ambrosioides* varied across the slag heap and wasteland sites, which indicated that the HM concentration of the soil may have some influence on the endophytic fungal diversity. Having better insight of total endophytic fungal community structure of the *D. ambrosioides* from HM contaminated sites could be useful in future studies to elucidate functional role of EF in the HM tolerance of the host plant.

## Conflict of interests

The authors declare no conflict of interests.
